# Genetic Factors Influence the Clustering of Depression among Individuals with Lower Socioeconomic Status

**DOI:** 10.1371/journal.pone.0005069

**Published:** 2009-03-31

**Authors:** Sandra López-León, Wing Chi Choy, Yurii S. Aulchenko, Stephan J. Claes, Ben A. Oostra, Johan P. Mackenbach, Cornelia M. van Duijn, A. Cecile J. W. Janssens

**Affiliations:** 1 Genetic-Epidemiology Unit, Department of Epidemiology & Biostatistics and Department of Clinical Genetics, Erasmus University Medical Center, Rotterdam, The Netherlands; 2 Department of Psychiatry, University of Leuven, Leuven, Belgium; 3 Department of Public Health, Erasmus University Medical Center, Rotterdam, The Netherlands; James Cook University, Australia

## Abstract

**Objective:**

To investigate the extent to which shared genetic factors can explain the clustering of depression among individuals with lower socioeconomic status, and to examine if neuroticism or intelligence are involved in these pathways.

**Methods:**

In total 2,383 participants (1,028 men and 1,355 women) of the Erasmus Rucphen Family Study were assessed with the Center for Epidemiologic Studies Depression Scale (CES-D) and the Hospital Anxiety and Depression Scale (HADS-D). Socioeconomic status was assessed as the highest level of education obtained. The role of shared genetic factors was quantified by estimating genetic correlations (ρG) between symptoms of depression and education level, with and without adjustment for premorbid intelligence and neuroticism scores.

**Results:**

Higher level of education was associated with lower depression scores (partial correlation coefficient −0.09 for CES-D and −0.17 for HADS-D). Significant genetic correlations were found between education and both CES-D (ρG = −0.65) and HADS-D (ρG = −0.50). The genetic correlations remained statistically significant after adjusting for premorbid intelligence and neuroticism scores.

**Conclusions:**

Our study suggests that shared genetic factors play a role in the co-occurrence of lower socioeconomic status and symptoms of depression, which suggest that genetic factors play a role in health inequalities. Further research is needed to investigate the validity, causality and generalizability of our results.

## Introduction

Depression is the leading cause of disability among individuals between 15 to 44 years of age, and is expected to become the second leading source of disability across all ages by 2020.[1] While depression occurs in individuals from all layers of our society, there is consistent evidence that the prevalence of depression is higher among individuals with lower socioeconomic status.[2]

The nature of the clustering between depression and socioeconomic status is multifactorial.[2], [3] Lower socioeconomic status may increase the risk of depression, as individuals from lower socioeconomic levels tend to have more stressful life events, poorer coping styles, and weaker social support networks, which put them at increased risk of developing depression.[4] Alternatively, depression may lead to lower socioeconomic status, for example depression in young adulthood may increase the risk of job loss, and lead to lower socioeconomic status and depression later in life.[3] Another hypothesis is that the clustering of depression and lower socioeconomic status is explained by shared causal pathways that lead to depression and lower socioeconomic status. For example, serotonin and dopamine pathways have been found to be involved in both traits and may be involved in both traits independently.[5], [6] Alternatively, low intelligence and neuroticism may share causal determinants as they are closely related to symptoms of depression and socioeconomic status. It has been known for long that neurotic persons are more vulnerable to depression,[7] and that persons with lower intelligence have a lower socioeconomic status.[7], [8] Further there is some evidence that higher levels of neuroticism are associated with lower academic performance,[9] and that depressed patients suffer from cognitive impairments.[10]

There has been interest to determine if genetic factors play a role in health inequalities. It has been proposed that for this to be possible, two conditions have to be met: (1) socioeconomic status has to be associated with one or more genotypes, and (2) those genotypes have to be themselves causally involved in the occurrence of health problems.[3] To date, only two genotypes have been studied in relation to socioeconomic status and genetic variants, but the results have not been reproduced. Two polymorphisms, one in the *DRD4* gene and the other in the *APOE* gene, have been studied for both depression and socioeconomic status.[11]–[14] When shared causal pathways are involved in the co-occurrence of depression and lower socioeconomic status, shared genes in these pathways may also be important.[15]

Both depression and socioeconomic status are partly determined by genetic predisposition. Heritability, which is the proportion of phenotypic variation in a population that is attributable to genetic variation among individuals, estimates range from 0.30 to 0.50 for socioeconomic status,[16]–[19] and from 0.17 to 0.78 for depression,[20] depending on the population investigated. The first aim of this study was to investigate the extent to which shared genetic factors can explain the clustering of depression among individuals with lower socioeconomic status. The second aim of the study was to examine if neuroticism or intelligence are involved in the clustering.

## Methods

### Ethics Statement N/A

#### Subjects

The present analyses were carried out using data from the Erasmus Rucphen Family (ERF) study. This family-based cohort study was designed to identify susceptibility genes for various complex disorders by studying quantitative traits. The ERF study is being conducted in a genetically isolated population located in the southwest of The Netherlands. The population is characterized by minimal immigration up until the last few decades. Genealogical information on this population was reconstructed using church and municipality records and is currently available in the form of a large database including over 63,000 individual records. In our analysis we included 2,383 individuals, with complete phenotypic and genealogical information was available.

Eligibility for participation in the study was determined by genealogical background, not by phenotypes of interest. Twenty-two families were selected who had at least six children baptized in the community church between 1880 and 1900. All living descendants of these families aged 18 years and older, as well as their spouses, were invited to attend a series of clinical examinations. Data were collected between June 2002 and February 2005. A detailed characterization of this population has been presented elsewhere.[21]–[23]

#### Procedures

All participants completed out questionnaires and underwent extensive medical examinations at the research center. The examinations were done by physicians of the academic center according to a standardized research protocol. Level of education, symptoms of depression, personality and premorbid intelligence were ascertained by questionnaires. Each participant completed the questionnaire once in the study period. Participants were asked to bring all medication to the research center and the use of antidepressant medication was verified by the physician. The research adhered to the tenets of the Declaration of Helsinki and was approved by the Medical Ethics Committee of Erasmus Medical Center in Rotterdam. Informed written consent was obtained after explanation of the nature and possible consequences of the study.

#### Measurements

Symptoms of depression were assessed using the Center for Epidemiologic Studies Depression Scale (CES-D),[24] and the depression subscale of the Hospital Anxiety Depression Scale (HADS-D).[25] Both scales are valid and reliable self-report measures of symptoms of depression.[26] The CES-D consists of 20 items with total scores ranging from 0 to 60, and the HADS-D of 7 items with scores ranging from 0 to 21. Higher scores indicate more symptoms of depression.

Socioeconomic status was assessed as the highest level of education obtained.[2] Seven education levels were distinguished and ranked from one (unfinished elementary, grade or primary school) to seven (college or university). Premorbid intelligence was assessed using the validated Dutch Adult Reading Test (DART).[27] DART premorbid intelligent scores range from 0 to 100 with higher scores indicating higher levels of premorbid intelligence. Neuroticism was measured using the NEO five factor inventory (NEO-FFI), a validated self-report questionnaire addressing five core personality traits: neuroticism, extraversion, openness, conscientiousness and agreeableness.[28] The NEO-FFI neuroticism scale consists of 12 statements with total scores ranging from 0 to 48. Higher scores indicate higher levels of neuroticism.

#### Statistical analysis

General characteristics were compared between men and women and tested using ANOVA for continuous variables and chi-squared test for dichotomous variables. To quantify the strength of the phenotypic association between symptoms of depression and education, partial correlations (ρ) were calculated. Associations were explored by univariate and multivariate linear regression (SPSS version 11.0 for Windows; SPSS, Chicago, IL). All determinants below the 0.10 significance level in the multivariate analyses were retained in the final model for heritability estimation. Multiple linear regression models were fitted to examine the association of covariates with symptoms of depression and to assess the distributional assumption of normality. The normality of residuals was tested using a one-sample Kolmogorov-Smirnov test. SPSS 11.0 for Windows was used.

A full pedigree variance components approach based on maximum-likelihood methods was used to estimate the heritability of symptoms of depression and of education.[29] Univariate quantitative genetic analysis was performed to partition the phenotypic variance of symptoms of depression variables into additive genetic and environmental variance components using maximum-likelihood variance decomposition methods.[30], [31] The phenotypic variance of the variables, which reflects the inter-individual variation, was partitioned into its additive genetic (σ^2^G) and residual environmental (σ^2^E) variance components.[32] The environmental variance is the mean residual, unexplained variance, which is not explained by the factors measured in the analysis (i.e. additive genetic factors or covariates). With genetic variance we mean the additive genetic component of the variance.

Heritability was estimated as the ratio of the additive genetic variance to the sum of the additive genetic and environmental variance, that is including sources of residual variance as measurement error: h^2^ = (additive) σ^2^G/(σ^2^G+σ^2^E). Dominance variance, which, in conjunction with additive and environmental variance, comprises broad sense heritability, was not estimated. Dominance effects are more easily modeled in twin than in family studies but they are difficult to model in extended pedigrees, we assumed additive effects.

Bivariate analyses were performed to estimate the genetic and environmental correlations between the symptoms of depression and education.[33], [34] The genetic and environmental correlations can be calculated from the phenotypic correlations (ρ_P_) by the following formula ρ_P_ = [square root]h_1_^2^[square root]h_2_^2^ρ_G_+[square root](1−h_1_^2^)[square root](1−h_2_^2^)ρ_E_,[35], [36] where h_1_^2^ and h_2_^2^ are the heritability estimates of the traits for which the phenotypic correlation is calculated, and ρ_G_ and ρ_E_ are the genetic and environmental correlations between these two traits. Significance of the phenotypic, additive genetic and environmental correlations was determined using a likelihood ratio test. To test whether a given correlation between two traits was significantly different from zero, the likelihood of a model in which this correlation was constrained to zero was compared with a model in which the same correlation was estimated. Twice the difference in ln-likelihoods of these models yields a test statistic that is asymptotically distributed as a chi-squared statistic with degrees of freedom equal to the difference in number of parameters estimated in the two models.

Analyses were adjusted for age, sex, use of medication, degree of consanguinity and sibship effects. The degree of consanguinity, indicating the degree to which parents of each participant are related to each other through their ancestors, was estimated using the Fortran software Package for Pedigree Analysis (PEDIG),[37] based on the pedigree of the total population. PEDIG yielded a coefficient for each participant, which was then entered as a covariate in the calculation of the heritability and genetic correlations. Sibship effects denote the exposure to early environmental factors that are shared by children of the same household.[33] In this study, sibship effect estimates were phenotypic similarities induced in the progeny of the same mother. This effect is a combination of effects induced by shared early life environment and dominant genetic effects. Because of the small number of half sibs in our sample and the non-delineation of household effects in our data set, the effect due to sharing the same mother is almost indistinguishable from the sibship effect.

Finally, to investigate the extent to which neuroticism and intelligence were intermediate factors in the causal pathway between the shared genetic factors and the co-occurrence of symptoms of depression and lower socioeconomic status, the analyses were additionally adjusted for NEO-FFI neuroticism scores and DART premorbid intelligence scores. In this analysis we assume that if neuroticism and intelligence are intermediate factors in the pathway, (genetic) correlations will disappear when adjusting for these factors. SOLAR (Sequential Oligogenic Linkage Analysis Routines) 2.1.2 software package (Southwest Foundation for Biomedical Research, San Antonio, Texas, USA) was used for the calculation of heritability estimates and for the genetic and environmental correlations. *P* values lower than 0.05 (two-tailed) were considered statistically significant.

## Results

The present analyses were based on data from 2,383 participants for whom complete phenotypic and genealogical information was available. Mean age of the participants was 48.7 years (SD 15.1) and 56.9% were women. Women reported more symptoms of depression on the CES-D scale and had higher NEO-FFI neuroticism scores (Table 1). Nine percent of the women reported the use of antidepressants compared to 4.3% of the men (p<0.001). Higher levels of education were associated with lower scores on the CES-D (ρ = −0.09, p<0.001) and HADS-D (ρ = −0.17, p<0.001; Table 2) scales. Higher DART premorbid intelligence scores were significantly correlated with higher levels of education (ρ = 0.49, p<0.001), and higher NEO-FFI neuroticism scores were significantly associated with higher scores on both scales of symptoms of depression scales (CES-D ρ = 0.51, p<0.001; HADS-D ρ = 0.50, p<0.001).

**Table 1:** General Characteristics of the Study Population.

	Men	Women	p-Value
	(n = 1,028)	(n = 1,355)	
Age, years	48.8 (14.7)	48.6 (15.3)	0.79
Education			
Lower	34.1	30.5	<0.001
Intermediate	58.1	65.8	
Higher	7.8	3.7	
Symptoms of depression			
CES-D scores	9.1 (8.6)	11.9 (10.2)	<0.001
HADS-D scores	6.0 (4.1)	6.1 (4.5)	0.62
Use of antidepressant medication	4.3	9.0	<0.001
DART premorbid intelligence scores	61.5 (19.4)	58.6 (19.2)	0.002
NEO-FFI neuroticism scores	29.7 (7.6)	32.6 (8.0)	<0.001

CES-D = Center for Epidemiologic Studies Depression Scale, DART = Dutch Adult Reading Test, HADS-D = Hospital Anxiety and Depression Scale - Depression subscale, NEO-FFI = NEO Five Factor Inventory.

Values are means (standard deviations) for continuous variables and percentages for categorical variables. *P*-values were obtained using χ^2^-statistics for categorical variables and univariate analysis of variance for continuous variables.

**Table 2:** Phenotypic Correlations Between the Study Variables.

	CES-D	HADS-D	Education	Intelligence
HADS-D	0.52***			
Education	−0.09***	−0.17***		
Education, adjusted for intelligence	−0.06**	−0.10**		
Education, adjusted for neuroticism	−0.02	−0.12***		
DART premorbid intelligence scores	−0.07**	−0.16***	0.49***	
NEO-FFI neuroticism scores	0.51***	0.51***	−0.15***	−0.15***

CES-D = Center for Epidemiologic Studies Depression Scale, DART = Dutch Adult Reading Test, HADS-D = Hospital Anxiety and Depression Scale - Depression subscale, NEO-FFI = NEO Five Factor Inventory.

*p<0.05, **p<0.01, *** *p*<0.001.

Values are partial correlations adjusted for age, sex, use of antidepressant medication.

Heritability estimates were 0.24 and 0.22 for the CES-D and HADS-D scores, 0.36 for education level, 0.54 for DART premorbid intelligence scores and 0.28 for NEO-FFI neuroticism scores. The heritability estimate of education decreased to 0.15 (p<0.001) after adjusting for DART premorbid intelligence scores, and the heritability estimates of CES-D and HADS-D depression scores decreased after adjusting for neuroticism (CES-D to 0.11 (p<0.001) and HADS-D to 0.12 (p<0.001).

Significant negative genetic correlations were found between education and both symptoms of depression scales (CES-D: ρG = −0.65, p<0.001; HADS-D: ρG = −0.50; p<0.001; Table 3). Genetic correlations between symptoms of depression and both DART premorbid intelligence scores and NEO-FFI neuroticism scores were statistically significant. The genetic correlations between education and symptoms of depression scores remained unchanged and statistically significant after additional adjustment for DART premorbid intelligence and NEO-FFI neuroticism scores (data not shown). The environmental correlation was statistically significant for the association of education level with CES-D scores (ρE = 0.10, p = 0.05) but not with HADS-D scores (ρE = 0.01, p = 0.89), and for the association of NEO-FFI neuroticism scores with CES-D and HADS-D scores (ρE = 0.51, p<0.001 and ρE = 0.42, p = 0.001).

**Table 3:** Genetic and Environmental Correlations Between Symptoms of Depression and Education, Intelligence and Neuroticism.

	CES-D		HADS-D	
	ρG	ρE	ρG	ρE
Education	−0.65 (0.14)***	0.10 (0.05)*	−0.50 (0.13)***	0.01 (0.05)
DART premorbid intelligence scores	−0.31 (0.11)***	0.04 (0.06)	−0.45 (0.11)***	0.04 (0.06)
NEO-FFI neuroticism scores	0.88 (0.06)***	0.51 (0.03)***	0.77 (0.10)***	0.42 (0.04)***

CES-D = Center for Epidemiologic Studies Depression Scale, DART = Dutch Adult Reading Test, HADS-D = Hospital Anxiety and Depression Scale - Depression subscale, NEO-FFI = NEO Five Factor Inventory. ρG = genetic correlation, ρE = environmental correlation.

*p<0.05, **p<0.01, *** *p*<0.001.

Analyses are adjusted for age, sex, use of antidepressant medication, degree of consanguinity and sibship effects. Values are genetic correlations with standard errors.

## Discussion

Our study demonstrates significant genetic correlations between symptoms of depression and level of education, suggesting that shared genetic factors play a role in the clustering of depression among individuals with lower socioeconomic status. This genetic correlation most likely reflects shared causal pathways with genetic factors that play a role in both depression and socioeconomic status. We further showed that these shared genetic pathways did not involve neuroticism or intelligence, as the genetic correlations remained unchanged after adjusting for these personality traits.

Before interpreting the findings, some issues should be addressed. First, we used education level as a proxy of socioeconomic status. Education is the most frequently used proxy, but this may be less appropriate in a three-generation study. Older participants with lower education levels may have acquired higher socioeconomic status by their work history, whereas younger participants have not yet had this opportunity. Hence, education level may be a less suitable proxy for socioeconomic status in older participants. Second, in our study symptoms of depression were assessed using two self-report questionnaires (CES-D and HADS-D). While the use of self-report questionnaires is widely accepted in epidemiological studies, self-report scales do have their limitations. Items and answer scales differ between the two depression scales and these may lead to different inferences about the depressive status of individuals. They may also explain the slight differences in the genetic and environmental correlations for the two scales that were observed in our analyses. Third, there is a possibility of potential interactions which were not taken into account in the analysis. For example, women in our population had a lower education and more symptoms of depression than men, but we did not test whether genetic correlation was greater in women. Fourth and last, we observed slight differences in the results obtained for the CES-D and the HADS-D scores. While both scales are validated for the assessment of symptoms of depression,[26] the scales differ in the items included and may therefore lead to different results. Note that while the estimates of the genetic correlations differed in magnitude, the overall pattern of association was the same for the CES-D and the HADS-D scales.

Our study focused for a large part on the co-occurrence of depression with other traits. This is a unique analysis that can be performed in family-based studies. By adjusting for sibship effects in our analysis we are taking into account the exposure to early environmental factors that are shared by children of the same household. So we are left with only the genetic effect present in familial clustering. With this method we were able to distinguish genetic effects from shared early environment effects to estimate a true genetic effect. However, we have to take into account that this estimate might be underestimated because sibship effects not only denote shared early environment, but also genetic dominance.

Education, which was used in our study as a proxy of socioeconomic status, is often viewed of as a purely environmental factor, but our study and that of others shows that genetic factors play a substantial role. The heritability estimate in our study was in line with previous studies that reported heritability estimates for education ranging from 0.30 to 0.50.[16]–[20] In our study the heritability of education was even higher than that of symptoms of depression. To date, only a few studies have assessed the association between education and genetic variants. Candidate genes which have been studied for both depression and education are the *DRD4* gene and the *APOE* gene.[11]–[14] However, for education no clear evidence has been established as the findings of genetic association studies have not yet been reproduced.

The negative genetic correlations between education level and CES-D and HADS-D suggest that the same underlying genetic factors lead to more symptoms of depression and lower socioeconomic status. We investigated whether intelligence or neuroticism were intermediate factors in this genetic pathway, but found no evidence for this hypothesis. Genetic factors that predispose to intelligence do contribute to the heritability of education, and predisposing genes for neuroticism contribute to the heritability of depression, but they do not explain the co-occurrence of symptoms of depression and lower socioeconomic status.

Improving health through the reduction of socioeconomic inequalities has been a public health goal for decades.[2] Depression is among other disorders, such as cardiovascular disease, that are consistently associated to low socioeconomic status.[2] Our results show that the co-occurrence may be partly explained by shared genetic factors, and suggest that genotypes may play a role in explaining health inequalities. Further research is needed to investigate the validity, causality and generalizability of our results. Most likely the depression is the consequence of a complex interaction between genes and environment. Developing programs to promote educational achievement and coping with life stresses in genetically vulnerable people will remain crucial.
